# Design and Implementation of a Real-time Monitoring Platform for Optimal Sepsis Care in an Emergency Department: Observational Cohort Study

**DOI:** 10.2196/26946

**Published:** 2021-06-24

**Authors:** Andy Hung-Yi Lee, Emily Aaronson, Kathryn A Hibbert, Micah H Flynn, Hayley Rutkey, Elizabeth Mort, Jonathan D Sonis, Kyan C Safavi

**Affiliations:** 1 Department of Emergency Medicine Massachusetts General Hospital Boston, MA United States; 2 Department of Medicine Massachusetts General Hospital Boston, MA United States; 3 Department of Anesthesia, Critical Care, and Pain Medicine Massachusetts General Hospital Boston, MA United States

**Keywords:** electronic monitoring platform, sepsis, quality improvement

## Abstract

**Background:**

Sepsis is the leading cause of death in US hospitals. Compliance with bundled care, specifically serial lactates, blood cultures, and antibiotics, improves outcomes but is often delayed or missed altogether in a busy practice environment.

**Objective:**

This study aims to design, implement, and validate a novel monitoring and alerting platform that provides real-time feedback to frontline emergency department (ED) providers regarding adherence to bundled care.

**Methods:**

This single-center, prospective, observational study was conducted in three phases: the design and technical development phase to build an initial version of the platform; the pilot phase to test and refine the platform in the clinical setting; and the postpilot rollout phase to fully implement the study intervention.

**Results:**

During the design and technical development, study team members and stakeholders identified the criteria for patient inclusion, selected bundle measures from the Center for Medicare and Medicaid Sepsis Core Measure for alerting, and defined alert thresholds, message content, delivery mechanisms, and recipients. Additional refinements were made based on 70 provider survey results during the pilot phase, including removing alerts for vasopressor initiation and modifying text in the pages to facilitate patient identification. During the 48 days of the postpilot rollout phase, 15,770 ED encounters were tracked and 711 patient encounters were included in the active monitoring cohort. In total, 634 pages were sent at a rate of 0.98 per attending physician shift. Overall, 38.3% (272/711) patients had at least one page. The missing bundle elements that triggered alerts included: antibiotics 41.6% (136/327), repeat lactate 32.4% (106/327), blood cultures 20.8% (68/327), and initial lactate 5.2% (17/327). Of the missing Sepsis Core Measures elements for which a page was sent, 38.2% (125/327) were successfully completed on time.

**Conclusions:**

A real-time sepsis care monitoring and alerting platform was created for the ED environment. The high proportion of patients with at least one alert suggested the significant potential for such a platform to improve care, whereas the overall number of alerts per clinician suggested a low risk of alarm fatigue. The study intervention warrants a more rigorous evaluation to ensure that the added alerts lead to better outcomes for patients with sepsis.

## Introduction

### Sepsis Background

Sepsis is the leading cause of death in US hospitals [1], accounting for 6% of all hospitalizations and 35% of all inpatient deaths [2]. Hospital performance in sepsis care has a significant impact on patient outcomes. A recent study demonstrated that every hour of delay in the completion of a sepsis bundle, including antibiotic administration, was associated with a 4% increase in risk-adjusted hospital mortality [3]. Thus, every failure to complete a sepsis bundle on time potentially represents a barrier to optimal patient care. There is a clear and urgent need for effective interventions to improve the performance of sepsis care and meet the expectations of patients and stakeholders.

International guidelines and the Centers for Medicare and Medicaid Services (CMS) Sepsis Core Measures (SEP-1) bundle emphasize the importance of adhering to specific steps in the diagnosis and management of sepsis [4]. Consistent adherence can be very challenging, especially in the setting of a busy emergency department (ED), ward, or intensive care unit, where there are multiple simultaneous demands on providers’ attention. In this environment, SEP-1 bundle care can be delayed or missed, even when team members are knowledgeable about best practices in sepsis care [5].

The CMS SEP-1 severe sepsis bundle is an all-or-nothing measure that requires antibiotics, blood cultures, and a lactate measurement within 3 hours of sepsis onset and a repeat lactate measurement within 6 hours if the initial lactate level is elevated. In cases of septic shock, additional requirements include adequate fluid resuscitation, reassessment of volume status and perfusion, and possible addition of vasopressors. As an all-or-nothing measure, a deficiency in any element of the bundle counts as failing the entire measure. In the first 2 years after the measure was introduced in 2015, two-thirds of sepsis cases submitted to CMS by hospitals failed the measure [6], and recent Hospital Compare data from 2019 still show a failure rate of 61% [7]. There has been argument in the literature about the appropriateness of some components of the bundle [8-10]; however, the fact remains that early identification and treatment of patients with sepsis remains an important quality opportunity.

### Strategies to Improve Sepsis Care

Institutions have attempted both nondigital and digital processes to improve sepsis care, including provider and nursing huddles and standardized electronic order sets. Most digital initiatives studied thus far have focused on identifying patients at risk for sepsis using the severity of illness scores in the electronic medical record (EMR). This has helped improve the early detection of sepsis [11,12] but has only modest effects on adherence to evidence-based bundle care elements and outcomes [13]. In concept, digital solutions offer the potential to close this gap by enabling real-time monitoring of adherence and providing just-in-time alerts to providers at the bedside. Electronically available data from the patient’s medical record would make this possible, as it includes most of the key elements of the SEP-1 bundle, including venipuncture for laboratory studies and medication administration. Any solution that could increase adherence and decrease cognitive load must also ensure that it does not cause alarm fatigue.

To date, we have not found any studies in the literature describing electronic alerting based on the completion of bundle elements for sepsis. Therefore, we developed a digital software solution called the Sepsis Care Tracking Platform (SCTP), which continuously monitors patients at risk of sepsis for completion of SEP-1 bundle elements and delivers actionable reminders to medical providers at the bedside in time for them to correct these deficiencies.

### Study Objectives

The objective of this paper is to describe the design, implementation, and validation of a novel electronic monitoring platform for optimal sepsis care in an ED at a large urban teaching hospital. By establishing feasibility, we hope to lay the groundwork for future randomized controlled studies that can determine the efficacy of the electronic monitoring platform in impacting process and outcome measures in sepsis care.

## Methods

### Initial Stakeholder Consultation

Multiple stakeholder groups were consulted to devise and implement the SCTP in the ED. For project approval and scope, the study team met with the chairperson and operational leaders of the Department of Emergency Medicine. The project was also presented to the quality and safety committees, both at the departmental and hospital levels. For feedback and iterative improvement, the ED Quality and Safety Committee was updated every 2 weeks regarding the project status and areas for improvement. ED providers who were paged by the SCTP received surveys regarding their experience and suggestions for improvement. This study was a quality improvement project based on institutional guidelines from the institutional review board and was thus exempted from the institutional review board review.

### Project Overview

The timeline for this project was divided into three phases: design and technical development, pilot, and postpilot program rollout.

#### Design and Technical Development Phase

The design and technical development phase spanned from September 1 to November 30, 2019. During this phase, the study team aimed to develop components of the SCTP and alert workflow, including defining the patient cohort, selecting the bundle measures from CMS SEP-1 for alerting, and determining recipients of alerts, mode of alert delivery, and alert content. In addition, technical development was performed, including linking EMR data to the platform, validating that the correct patients and bundle elements were captured, and performing initial quality control on data and alerts via chart review.

#### Pilot Phase

The pilot phase spanned from December 9, 2019, to January 12, 2020. During this phase, the study team deployed the initial version of the platform in the ED, including monitoring actual patients and sending alerts to their providers. This phase aimed to ensure the technical stability of the platform through confirmed receipt of alerts, monitor for false-positive results, and obtain feedback from alert recipients via a survey to determine the usefulness of the platform, alarm fatigue, and potential improvements. Monitoring for false positives was performed by study team members who manually verified every page sent during the pilot phase by confirming through the patient record that alerts reflected the correct bundle element deficiencies. Providers who received a page would be emailed a two-question survey the next day asking for feedback regarding each page. The two questions in the survey were (1) *Was this page alert useful in providing sepsis care to the patient?* with a ranking from *not at all helpful* (1) to *extremely helpful* (5), and (2) *If not helpful, why?* An additional space allowed for additional feedback, and providers could receive the survey multiple times for different pages.

#### Postpilot Rollout Phase

After incorporating modifications and improvements identified during the pilot phase, the postpilot rollout was initiated, in which a prospective trial was performed between January 13, 2020, and March 1, 2020. This trial aimed to assess the function of the platform and describe alerting patterns and subsequent adherence to alerts during this period.

### Platform Setup

The SCTP ED platform was coded in C#, using .NET Microsoft Development Platform and Microsoft SQL databases. It was linked to the institutional EMR (EPIC) through a web service that extracted data each minute. The SCTP checked for the completion of the sepsis bundle elements every 15 minutes. These elements included completion of blood culture orders; initial and repeat lactate results; and administration of antibiotics, vasopressors, and fluids (as described in the *Results* section, vasopressors and fluids were later eliminated from the SCTP). If one or more elements were incomplete, the ED providers would be paged with the deficiency. Each patient was added to the platform after the institutional sepsis best practice alert (BPA) was accepted and monitored until the end of the 6-hour window for optimal sepsis care. The platform tracked a patient within that window even if the patient was admitted or transferred to a different area of the hospital and continued to page the appropriate responding clinician.

### Study Measures and Analysis

During both the pilot and postpilot program rollout phases, several study measures were tracked to assess the platform function and outcomes. Data were abstracted from the SCTP, an institutional clinical and administrative database, and surveys administered to paged providers. Subsequent data analyses were performed using Microsoft Excel.

We defined an *alert* as when the SCTP identified a sepsis bundle element deficiency within 1 hour of the bundle element becoming due. We defined a *page* as occurring when a clinical provider received a notification on their pager. As there were usually multiple providers caring for each patient, a single *alert* could trigger multiple *pages* at the same time. During the pilot phase, we assessed the total number of pages sent to providers; the number of alerts per provider shift, defined as the number of alerts divided by the number of shifts completed by the attending providers; and the etiology of alerts, defined as the bundle measure that triggered the alert. In addition, we administered an email survey to the providers after receiving an alert. The survey consisted of two questions regarding whether the pages were helpful and whether they had any further feedback regarding the SCTP.

During the postpilot program rollout, we measured the characteristics of patients who were monitored by the platform, including age and length of stay in the ED. Similar to the pilot phase, we also measured the number and etiology of the pages sent to the providers. In addition, we measured the *postalert successful adherence rate* of sepsis bundle measures, which was defined as the completion of the sepsis bundle element within the next hour after the relevant page was sent to the provider.

## Results

### SCTP Design and Technical Development Phase

The SCTP was developed and validated in close cooperation with ED Quality and Safety leadership and intended to fit within the existing workflow of the institution to decrease barriers to adoption.

### Inclusion Criteria for Active Monitoring

**Figure 1:**
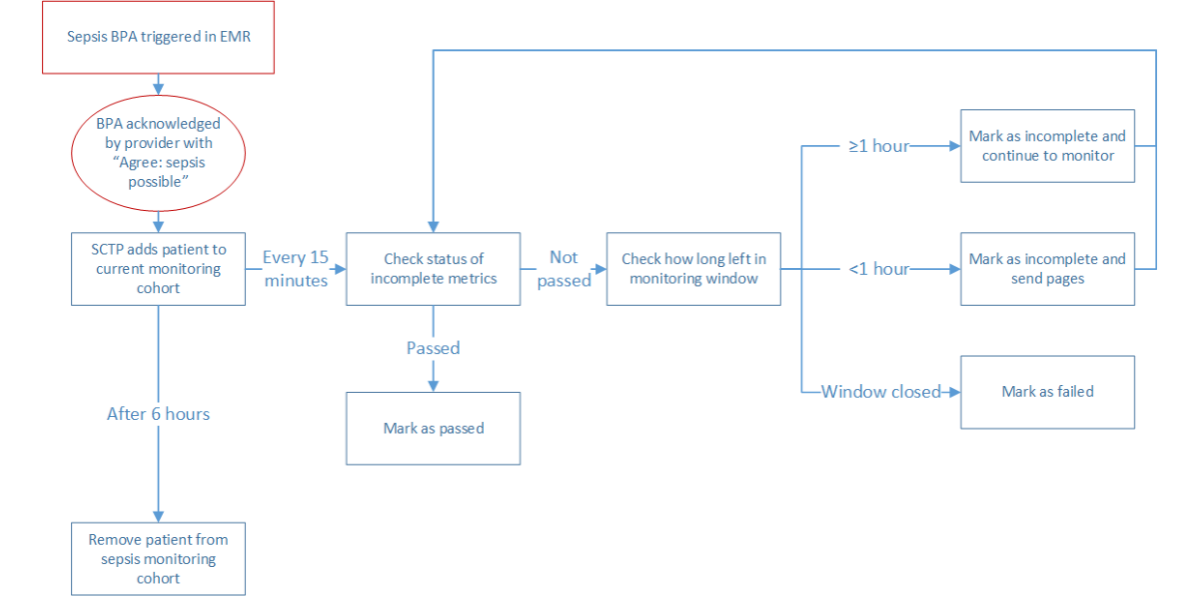
Sepsis care tracking platform page generation logic. BPA: best practice alert; EMR: electronic medical record; SCTP: Sepsis Care Tracking Platform; SEP-1: sepsis core measure.

In consultation with ED Quality and Safety leadership, it was determined that patients should qualify for monitoring if they met the criteria for possible sepsis based on an electronic health record (EHR)–derived algorithm that existed before this study. This algorithm is based on the Sepsis-3 2016 European Society of Intensive Care Medicine and Society of Critical Care Medicine consensus definition of sepsis and incorporates both signs of infection such as fever, positive laboratory results, and patient risk factors, as well as signs of organ dysfunction as measured by the Sequential Organ Failure Assessment score [14]. Providers caring for patients identified as potentially septic receive a prompt in the EHR in which they could click that they agreed or disagreed that sepsis was likely. If accepted, the patient would qualify for SCTP monitoring. Stakeholders and study team members believed that the advantage of this approach was to include only those patients in the active monitoring cohort whom the team suspected of sepsis, thereby increasing the relevance of SCTP alerts they received (Figure 1).

### Measurement Selection for Alerting

From the commonly accepted SEP-1 bundle, stakeholders selected antibiotics, blood cultures, and lactate measurements as the bundle elements that would be monitored and excluded measures related to the volume of intravenous fluids administered. The decision to exclude fluids was made because of the belief that significant practice variation existed due to patient-specific conditions and comorbidities [15,16] and that alerts suggesting that additional fluids should be administered could be clinically inappropriate.

### Alert Time Threshold

Stakeholders agreed that paging 1 hour before the deadline for bundle compliance gave providers sufficient time to address the deficiency (Figure 1). To minimize alert fatigue, stakeholders agreed that providers would receive a maximum of two pages per patient that would be sent 1 hour before the 3-hour and 6-hour SEP-1 time windows. Each page would list all the deficient bundle elements at that time.

### Alert Recipients

**Figure 2:**
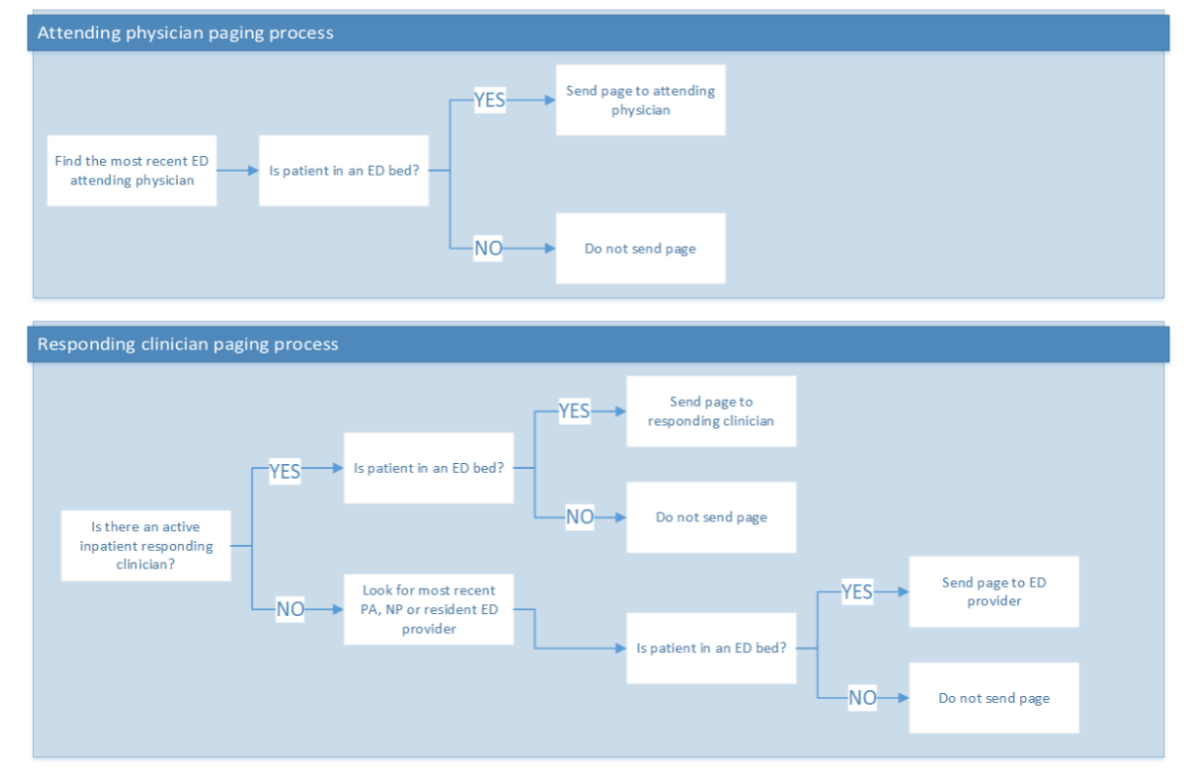
Sepsis care tracking platform page recipient logic. ED: emergency department; NP: nurse practitioner; PA: physician assistant.

Given that multiple providers were simultaneously responsible for a single patient, stakeholders designed the software to use the logic shown in Figure 2 to determine the appropriate clinicians to page. The decision was made to send alerts to both the responding clinician (resident or advanced practitioner) and the supervising attending because each played a unique role in the patient’s sepsis care. The resident and advanced practitioner were responsible for placing orders and closing the loop of communication with bedside nurses about bundle measures, whereas the attending physician could supervise and monitor the overall care of the patient to ensure appropriate care was provided. Although the study team and ED stakeholders thought it valuable to alert bedside nurses, given their important role in sepsis care delivery, this was technically not feasible as they did not carry pager devices.

### Alert Message Contents

**Figure 3:**
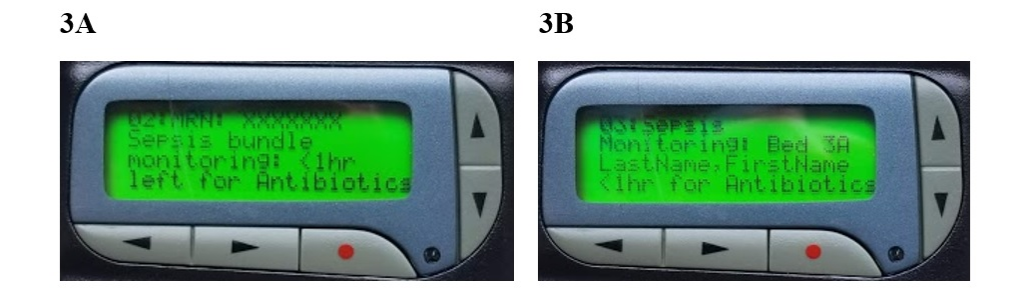
Pilot phase (3A) and rollout phase (3B) pager display.

Stakeholders agreed that alert message contents should state the deficient element without providing prescriptive language recommending that the provider performs a particular action (Figure 3). The message contents would list which element was deficient, but otherwise the remainder of the message would remain the same for all pages. This decision was made on the rationale that in some cases providers would appropriately not administer bundle care to patients based on the evolving diagnostic and clinical course of the patient.

### Alert Delivery Mechanism

Stakeholders determined that the hospital’s paging system was the most effective means of communicating time-sensitive alerts, given that providers always carry their pager and typically view messages immediately. This was preferred over EMR-based pop-up alerts because it required the provider to log on to the patient’s medical record to see the alerts. It was felt that this mode would be burdensome to the provider and potentially delay the receipt of the message.

### Pilot Phase

During the 35-day period, 453 patients met the inclusion criteria to be monitored by the SCTP. Overall, 371 pages were sent, resulting in 0.79 pages per attending shift. Of the alerts, 27.3% (50/183) were for blood cultures, 50.3% (92/183) were for antibiotics, and 22.4% (41/183) were for lactate measurements.

In total, 70 surveys were returned with responses. In total, 87% (61/70) of respondents found the alerts to be helpful (21/70, 30%), very helpful (17/70, 24%), or extremely helpful (23/70, 33%). Specific examples of positive feedback included “The page helped us realize blood cultures were not initially ordered and we ordered them after this page” and “It prompted us to reassess the patient for sepsis.” Providers also suggested that pages “would be more useful if patient’s name was also displayed on the page and not only the MRN.” Providers also noted that their suspicion for sepsis can change over the course of an ED visit, with comments such as “Patient was later felt not to have sepsis so bundle was not completed.” On the basis of this feedback, the alert message content was modified to allow providers to more easily identify the patient (Figure 3) and the alert routing algorithm was adjusted to identify the correct attending physician (Figure 2). We also removed reminders to initiate vasopressors for hypotensive patients because a reliable bedside monitoring alarm system already existed to alert clinicians to hypotension and clinicians consistently responded by initiating vasopressors when appropriate. Our survey responses helped to elucidate this frontline practice.

### Postpilot Program Rollout Phase

After completion of the pilot program with subsequent adjustments to the SCTP, the postpilot program rollout phase was initiated. During this 48-day period, 711 patient encounters (mean 14.8, SD 4.4 encounters per day) for 648 unique patients met the inclusion criteria to be monitored by the SCTP (Table 1).

Of the 711 encounters monitored by the SCTP, 439 (61.7%) passed all measures without requiring a page, as all sepsis bundle elements were completed more than an hour before the deadline. In comparison, 38.3% (272/711) patients had one or more pages sent to the responding providers. In total, 634 pages were sent, averaging 0.89 pages per patient in the active monitoring cohort and 0.98 pages per attending shift (Table 2).

Of the 634 pages, the most frequent alert sent was for deficient antibiotics (136/327, 41.6%), followed by the second lactate measurement (106/327, 32.4%), blood cultures (68/327, 20.8%), and first lactate (17/327, 5.2%). This distribution was similar to the results from the pilot study, with pages related to antibiotics being the most common etiology.

Overall, there was a substantial proportion of sepsis bundle element deficiencies that triggered a page and were successfully adhered to within the SEP-1 time window. There was a 26% (18/68) postalert successful adherence rate for collecting blood cultures, 12.5% (17/136) for administering antibiotics, and 24% (4/17) for initial lactate measurement and an impressive 81.1% (86/106) for the second lactate measurement.

**Table 1:** Postpilot rollout phase patient demographics (N=648).

Characteristics			Values, n (%)
**Age (years)**			
	18-65	271 (41.8)	
	>65	377 (58.2)	
**Sex**			
	Female	294 (45.4)	
	Male	354 (54.6)	
**ICU^a^** **admission**			
	No	468 (72.2)	
	Yes	180 (27.8)	
**Emergency department** **LOS^b^** **(hours)**			
	≤6	236 (36.4)	
	>6	339 (52.3)	

^a^ICU: intensive care unit.

^b^LOS: length of stay.

**Table 2:** Postpilot rollout phase paging and alert data.

Characteristics			Values
Emergency department encounters			15,770
Encounters in active monitoring (per day), mean (SD)			711 (14.8)
Total pages (per day), mean (SD)			634 (13.2)
Pages per attending shift			0.98
Encounters with at least one page (n=711), n (%)			272 (38.3)
Encounters with no pages (n=711), n (%)			439 (61.7)
**Alert etiology (n=327), n (%)**			
	Total	327 (100)	
	Blood cultures	68 (20.8)	
	Antibiotics	136 (41.6)	
	Initial lactate	17 (5.2)	
	Repeat lactate	106 (32.4)	
**Postalert successful adherence (n=327), n (%)**			
	Total	125 (38.2)	
	Blood cultures	18 (26.5)	
	Antibiotics	17 (12.5)	
	Initial lactate	4 (23.5)	
	Repeat lactate	86 (81.1)	

## Discussion

### Principal Findings

To improve adherence to sepsis guideline care at our tertiary care hospital, we designed and implemented a real-time sepsis care tracking and alerting platform in a busy ED environment. With feedback from frontline clinicians, quality and safety program representatives, and departmental leadership, we customized the SCTP to the needs of clinicians and the clinical environment. In our postpilot program rollout phase, the SCTP actively monitored 711 ED encounters where sepsis was thought to be likely, in 272 of these encounters, the platform sent text pages to ED providers due to a deficiency in sepsis bundle completion, with an overall postpage adherence rate of 38.2% (125/327).

During the development phase, it was important to select the sepsis bundle elements that were felt to be most clinically appropriate and could be identified by our monitoring platform. The SEP-1 framework served as a foundation for this study, as the framework was widely accepted and aligned the interests of multiple stakeholder groups, including providers, departmental and quality and safety leadership, and hospital administration. Engaging with provider stakeholders allowed us to make the application more effective. Through these discussions, we were able to understand the clinical workflow and target alerting to the frontline provider who places the orders.

Moreover, we note the importance of the provider feedback obtained during the pilot phase. The surveys emphasized the need to provide critical information concisely in the SCTP paging alerts, including what information can quickly and accurately identify the patient and the specific bundle element deficiency. In addition, given the complex structure of provider teams in our ED, which can change multiple times over a patient’s ED visit, we had to consider different clinical scenarios and which provider would be the optimal recipient for paging alerts. Finally, given the learning curve that comes with new technologies, the pilot phase allowed providers to acclimate to this system before the full rollout.

During the postpilot rollout, we found that there was a substantial proportion of encounters (272/711, 38.3%) in which the clinician was at risk of missing the SEP-1 care measure and for which an alert was paged, indicating the potential impact of such a platform on adherence. Furthermore, we found that the postalert successful adherence rate ranged from 12.5% (17/136) to 81.1% (86/106), depending on the measure for which the alert was sent, further suggesting the potential for the platform to improve adherence. Finally, we found that the number of pages per clinician shift (0.98) was low enough to not be overly disruptive but high enough that we would expect the SCTP to meaningfully impact SEP-1 performance. On the basis of these findings, we believe that a real-time sepsis care tracking and alerting platform is feasible in a hectic ED environment and could have a significant impact on adherence to life-saving sepsis care without causing alarm fatigue.

In addition, we found it concerning that the most common deficiency triggering a paging alert was a delay in antibiotics, which is the SEP-1 element that is most closely linked to mortality in previous studies [17]. This suggests the possibility that the SCTP could be associated with mortality improvement if it is able to improve timely antibiotic administration. However, we found that the postalert adherence success rate for antibiotics was the lowest among all alerts. It may be that antibiotics were, in fact, administered just outside of the monitored 3-hour window or that patients were subsequently determined to likely not have sepsis. Regardless, this discrepancy requires further exploration to determine why antibiotics may have a lower rate of postalert adherence compared with other measures.

In comparison, we found that repeat lactate was the second most common etiology of pages (106/327, 32.4%) and had the highest postadherence rate (86/106, 81.1%). This suggests that the SCTP may be especially valuable over a prolonged time course in which providers may be distracted by the needs of other patients, and paging alerts in this situation can refocus provider attention with sufficient time to deliver optimal care.

We believe that the postalert adherence rate of 12.5% (17/136) to 81.1% (86/106) for bundle element deficiencies represents the completion of bundle elements that likely would have been missed otherwise and is a significant potential improvement in increasing adherence to sepsis bundle care. However, the majority of pages did not result in adherence, which deserves further study. On the basis of our survey data, we believe that an important driver of this trend may be that the clinician no longer believed sepsis was likely. The sepsis BPA, which the SCTP used as a trigger to begin monitoring, was designed to cast a wide net early in the patient’s ED encounter to reduce the likelihood of missing any patient who could be septic given the high mortality of the disease. As more data are gathered and the patient’s care evolves, clinical teams may have judged that sepsis was unlikely and thus appropriately withheld sepsis bundle care. We believe that this could have accounted for much of the nonadherence. A future iteration of the SCTP may enable frontline clinicians to *off ramp* from the page alerts if they judge that sepsis is highly unlikely later in the patient’s ED course.

SCTP differs from previous EHR-related innovations in improving sepsis care. The majority of previous studies have focused on identifying sepsis through different clinical criteria [18] or through predictive algorithms and machine learning [19], usually followed by an EHR warning or paging alert. Other studies have used alerts to trigger sepsis workflows, including bedside assessment and order sets [11]. SCTP builds on these innovations by following patients suspected of having sepsis through the next several hours of care, which can be the most critical and prompts providers toward optimal care. Therefore, we expect it to influence provider action more directly for patients suspected of having sepsis and have a meaningful impact on clinical outcomes.

The focus of this study is on alert fatigue, as recent articles have shown that an increase in the number of alerts decreases the provider’s acceptance rate [20] and many alerts in the EHR may not directly change clinical care [21]. The SCTP incorporated many design features to minimize alert fatigue, such as restricting enrollment to patients where the sepsis BPA was confirmed by an ED provider and condensing pages so no more than two pages are sent for any patient. Consultations with frontline providers and survey data were also used to optimize the message content to determine the appropriate page recipient. All of these factors likely contributed to the low number of pages sent per attending shift (0.98) and favorable survey responses. However, further work is needed to ensure that the clinical benefit of this system outweighs the alert fatigue it generates.

This study had several limitations. First, system implementation was conducted at a single academic site, which may not be generalizable across different health care settings. Second, the survey response rate was low for providers receiving alerts from the paging system. Subsequent iterations may consider text-based phone messaging or in-person surveys at the time of the provider receiving the page, which may encourage more detailed and complete responses. Third, the study was not designed to measure the effect size of SCTP paging alerts or review patient cases where providers understand sepsis to be unlikely despite the initial BPA. Our institution is currently initiating a randomized controlled trial to determine the efficacy of SCTP in improving compliance with SEP-1 bundle elements and patient outcomes. It will also further quantify any marginal increase in alert fatigue. Finally, there may be other simultaneous quality improvement initiatives and the Hawthorne effect that may confound our data.

### Conclusions

In conclusion, we have demonstrated a feasible, technically sound, real-time provider alert system that has the potential to improve the SEP-1 compliance rate without significant amounts of alert fatigue. We anticipate that future work will involve a randomized controlled trial to measure potential increases in SEP-1 compliance rates and patient outcomes and further characterize alert fatigue. The expansion of this promising intervention could be considered in other inpatient settings.

## Abbreviations

BPA: best practice alert
CMS: Centers for Medicare and Medicaid Services
ED: emergency department
EHR: electronic health record
EMR: electronic medical record
SCTP: Sepsis Care Tracking Platform
SEP-1: sepsis core measure
